# The association between patellar malalignment and Osgood–Schlatter disease in children

**DOI:** 10.3389/fped.2026.1753322

**Published:** 2026-03-13

**Authors:** Yang Huibao, Long Dawei, Wang Shuo, Kuang Xueao, Wei Shuailong, Wang Zhenzhen, Guo Tianxiang

**Affiliations:** 1 First Clinical Medical College, Anhui University of Chinese Medicine, Hefei, China; 2 The Seventh Affiliated Hospital, Anhui University of Chinese Medicine, Fuyang, China

**Keywords:** biomechanics, Insall–Salvati index, Osgood–Schlatter disease, patella alta, pediatric orthopedics

## Abstract

**Objective:**

The aim of this study was to investigate the association between patellar position [assessed by the Insall–Salvati index (ISI)] and Osgood–Schlatter disease (OSD) in children, and to evaluate the predictive value of ISI and body mass index (BMI) for OSD.

**Methods:**

A case–control/cross-sectional study was conducted enrolling 127 children aged 10–16 years between March 2023 and June 2025, including 76 children with OSD (observation group) and 51 healthy children (control group). ISI values were measured radiographically, and data on BMI, visual analog scale score, gender, and recent exercise level were collected. Univariate analysis and multivariate logistic regression were used to analyze associated factors, and the predictive performance of ISI and BMI was evaluated using receiver operating characteristic (ROC) curves.

**Results:**

The observation group had significantly higher ISI values, BMI values, and proportion of males compared to the control group (all *P* < 0.05). Multivariate logistic regression identified that the male gender (OR = 5.61), high BMI (OR = 1.77), high ISI (OR = 16.13), and recent high exercise level (OR = 7.03) were independent risk factors for OSD (all *P* < 0.05). ROC curve analysis indicated that the area under the curve (AUC) for ISI predicting OSD was 0.933 (optimal cutoff value 1.14), superior to the AUC for BMI (0.793).

**Conclusion:**

The ISI value is a strong independent indicator associated with OSD, with an optimal cutoff value of 1.14. The pathogenesis of OSD may involve the combined effects of abnormal biomechanics (high ISI) and mechanical load (high BMI and high exercise level). Early screening of ISI may help identify high-risk individuals for targeted prevention.

## 1 Introduction

Osgood–Schlatter disease (OSD) is a common overuse knee injury in adolescents, particularly prevalent among physically active individuals aged 10–16 years. The pathological basis of OSD lies in the susceptibility of the developing tibial tuberosity apophysis, which is not fully ossified, to local inflammation, injury, and even minor avulsion fractures under repetitive and intense traction from the quadriceps muscle (1). According to Wolff's Law, bone adapts to long-term mechanical stress through changes in density and structure, leading to local bone hyperplasia and tuberosity prominence, which can exacerbate clinical symptoms. Current research generally acknowledges that the pathogenesis of OSD involves multiple interacting factors, including quadriceps muscle dysfunction (2), alterations in patellar tendon mechanical properties (3), increased external mechanical load (4), variations in tibial anatomy (5), and changes in the composition of the epiphyseal cartilage (6). Notably, the patella, as a key structure in knee biomechanics, may influence stress distribution on the tibial tubercle through alterations in patellar tendon tension and quadriceps moment arm if its position is abnormal. The Insall–Salvati index (ISI), a classic radiographic parameter for assessing patellar position, has an unclear relationship with OSD risk. Based on preliminary clinical observations, we hypothesized that abnormal patellar position (particularly patella alta) and its corresponding ISI value might be associated with the occurrence of OSD. Therefore, this case–control study was designed to systematically analyze the correlation between ISI and OSD, and further explore the predictive value of ISI and body mass index (BMI) for this condition, with the aim of providing a theoretical basis for early identification and prevention of OSD.

## 2 Clinical data and methods

### 2.1 Clinical data

A total of 127 children (age 10–16 years, boys/girls: 79/38) admitted to our hospital between March 2023 and June 2025 were enrolled. Participants were divided into an observation group (76 children with OSD, further subdivided into patella alta, patella baja, and normal patella subgroups) and a control group (51 healthy children, 24 boys, 27 girls). The healthy children in the control group were identified through a retrospective review of past clinical cases (both outpatient and inpatient), and their lateral knee radiographs, originally obtained for clinical indications, were utilized for analysis. This study was approved by the hospital ethics committee.

### 2.2 Inclusion criteria

(1) The children were required to present with pain or swelling at the tibial tuberosity, limited knee movement, with radiographic or ultrasonographic examination showing enlargement, fragmentation, or free bone fragments of the tibial tuberosity apophysis. (2) The children had to be between 10 and 16 years of age. (3) Guardians and the children themselves were required to agree to participate in the study and provide signed informed consent.

### 2.3 Exclusion criteria

(1) Patients with concurrent fractures, bone tuberculosis, bone infections, etc.; (2) patients with severe impairment of heart, liver, or kidney function, or coagulation disorders; (3) patients with severe cardiovascular or cerebrovascular diseases; and (4) patients with comorbid mental illness were excluded from this analysis.

### 2.4 Measurement methods and observation indicators

Control group (healthy) children underwent lateral knee X-rays at 30° flexion. As this was a retrospective review, some images may not have been acquired at this exact standard position. In the observation group, the ISI was measured using the imaging tools available in their clinical records [digital radiography (DR), magnetic resonance (MR), or computed tomography CT)]. To assess measurement reliability, inter- and intraobserver analyses were performed. Two radiologists, blinded to the group allocation, independently measured ISI using system measurement tools. Interobserver reliability was good (intraclass correlation coefficient, ICC = 0.87, 95% CI: 0.75–0.94). For intraobserver reliability, one researcher repeated measurements on 15 randomly selected samples after 2 weeks, yielding excellent consistency (ICC = 0.92, 95% CI: 0.81–0.97). The average of the two initial measurements was used for analysis to reduce error. The visual analog scale (VAS) was used for pain assessment (score 0–10, higher score indicating greater pain). The child's gender and BMI were recorded.

Recent exercise level over the preceding week was categorized as low, normal, or high based on self-reported minutes of moderate-to-vigorous physical activity (MVPA), following World Health Organization recommendations. The specific criteria were as follows: low exercise level: MVPA < 150 min/week; normal exercise level: MVPA 150–300 min/week; and high exercise level: MVPA > 300 min/week.

### 2.5 Statistical analysis

Statistical analyses were performed using SPSS 26.0 software. Measurement data conforming to normal distribution were expressed as mean ± standard deviation (x¯ ± s) and analyzed using univariate analysis. Data not conforming to normal distribution were expressed as median (interquartile range) [*M* (Q1, Q3)] and compared using non-parametric tests (Mann–Whitney *U* test, rank test, *H* test). A *p* < 0.05 was considered statistically significant. Binary logistic regression was used to analyze the correlation between OSD onset and gender, exercise level, BMI, and ISI. Receiver operating characteristic (ROC) curve analysis was used to evaluate the predictive value of ISI and BMI for OSD onset.

## 3 Results

### 3.1 Comparison of gender, Age, ISI, BMI, and VAS scores between groups

No statistically significant difference was observed in age between the two groups (*P* > 0.05). However, gender composition significantly differed (*P* < 0.05). The control group had significantly lower ISI values, BMI values, and VAS scores compared with the observation group (*P* < 0.05), as shown in Table 1.

**Table 1:** Comparison of gender, age, ISI value, BMI value, and VAS score between the two groups.

Group	*n*	Age	Male/female	ISI value	BMI value	VAS score
Observation	76	13 (11, 14)	66/10	1.18 (1.15, 1.26)	24.6 (21.3, 25.1)	3 (2,3)
Control	51	13 (11, 15)	23/28	1.02 (0.90, 1.11)	20.5 (19.6, 21.5)	–
Statistic	–	−0.975	*χ^2^* *=* 23.36	*Z* = −7.46	*Z* = −5.59	*Z* = −10.134
*P*-value		*P* > 0.05	*P* < 0.05	*P* < 0.05	*P* < 0.05	*P* < 0.05

### 3.2 Comparison of patellar position between groups

The composition ratio of patellar position was statistically significant between the observation group and the control group (*P* < 0.05), as shown in Table 2.

**Table 2:** Comparison of patellar position between the Two groups.

Group	Patellar position		
	Alta	Baja	Normal
Observation	15	2	59
Control	2	1	48
Statistic	*χ^2^* *=* 6.75		
*P*-value	*P* < 0.05		

### 3.3 Multivariate logistic regression analysis of influencing factors for OSD onset

Multivariate analysis showed that age had no significant effect on OSD (*P* > 0.05), while gender, BMI, ISI, and exercise level were associated with OSD onset (*P* < 0.05), as shown in Table 3.

**Table 3:** Multivariate logistic regression analysis of influencing factors for OSD onset in children.

Variable	*β*	SE	Wald *χ^2^*	OR (95% CI)	*P*-value
Constant	−15.48	3.90	15.73	–	–
Gender (male)	1.73	0.64	7.21	5.61 (1.59, 19.77)	0.007
BMI value	0.57	0.17	11.31	1.77 (1.27, 2.47)	0.001
ISI value	2.78	0.64	18.92	16.13 (4.61, 56.47)	0.001
High exercise level (recent 1 week)	1.95	0.77	6.48	7.03 (1.57, 31.51)	0.011

### 3.4 Predictive value of ISI and BMI for OSD

The areas under the ROC curve for ISI and BMI were 0.933 and 0.793, respectively. The predictive value of ISI was greater than that of BMI (*P* < 0.05), as shown in Table 4.

**Table 4:** Predictive value of ISI and BMI for OSD.

Item	AUC (95% CI)	Cutoff value	Sensitivity (%)	Specificity (%)	Youden index
ISI	0.933 (0.883, 0.984)	1.14	92.1	94.1	0.862
BMI	0.793 (0.716, 0.871)	23.05 kg/m^2^	67.1	92.2	0.593

**Figure 1:**
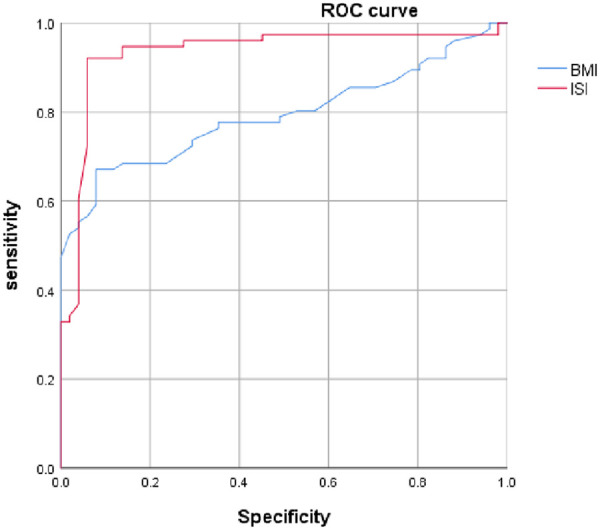
Receiver operating characteristic curves of ISI and BMI for predicting Osgood–Schlatter disease in children.

Figure 1 shows the ROC curves for ISI and BMI in predicting pediatric OSD. The predictive performance of ISI is superior to that of BMI.

**Figure 2:**
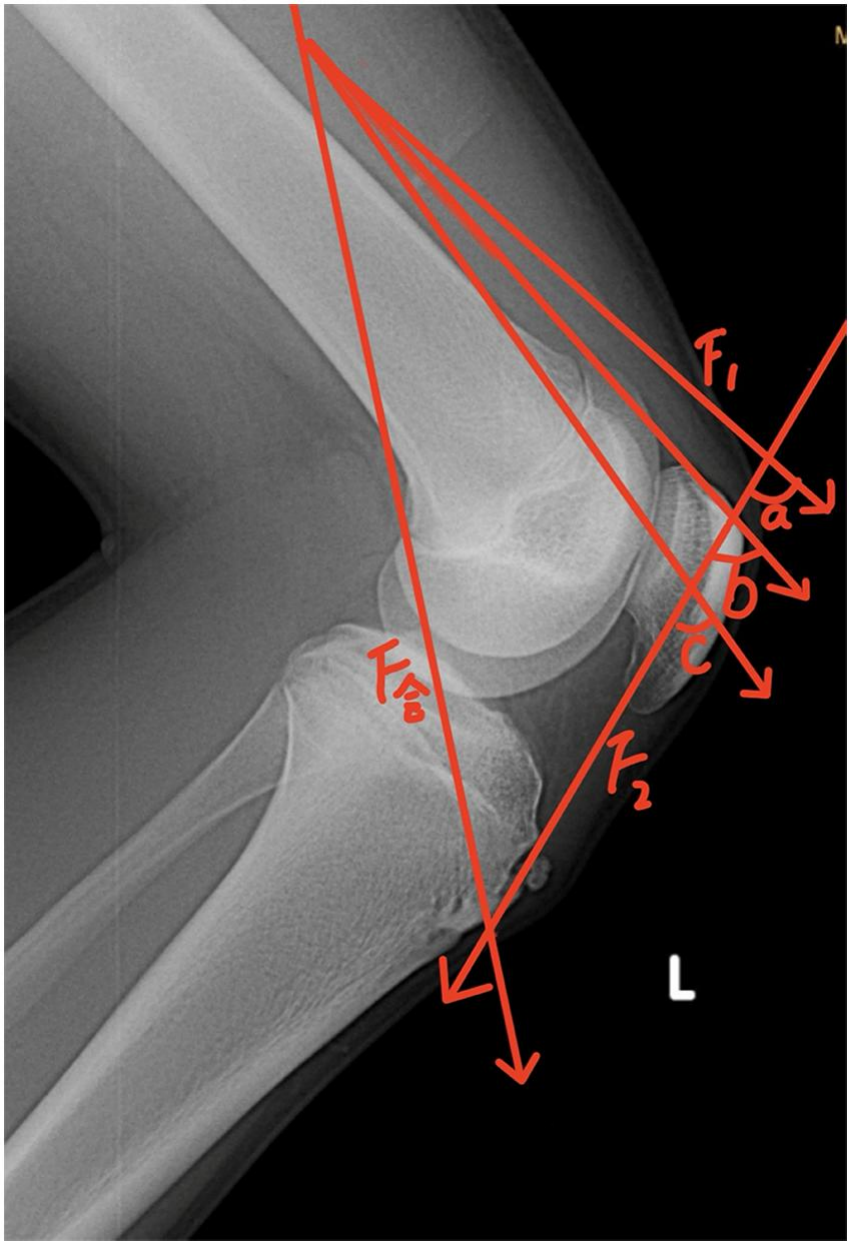
Biomechanical force vector analysis of the tibial tuberosity apophysis in OSD patients: X-ray of the flexed knee joint with labeled force components and relevant angles.

**Figure 3:**
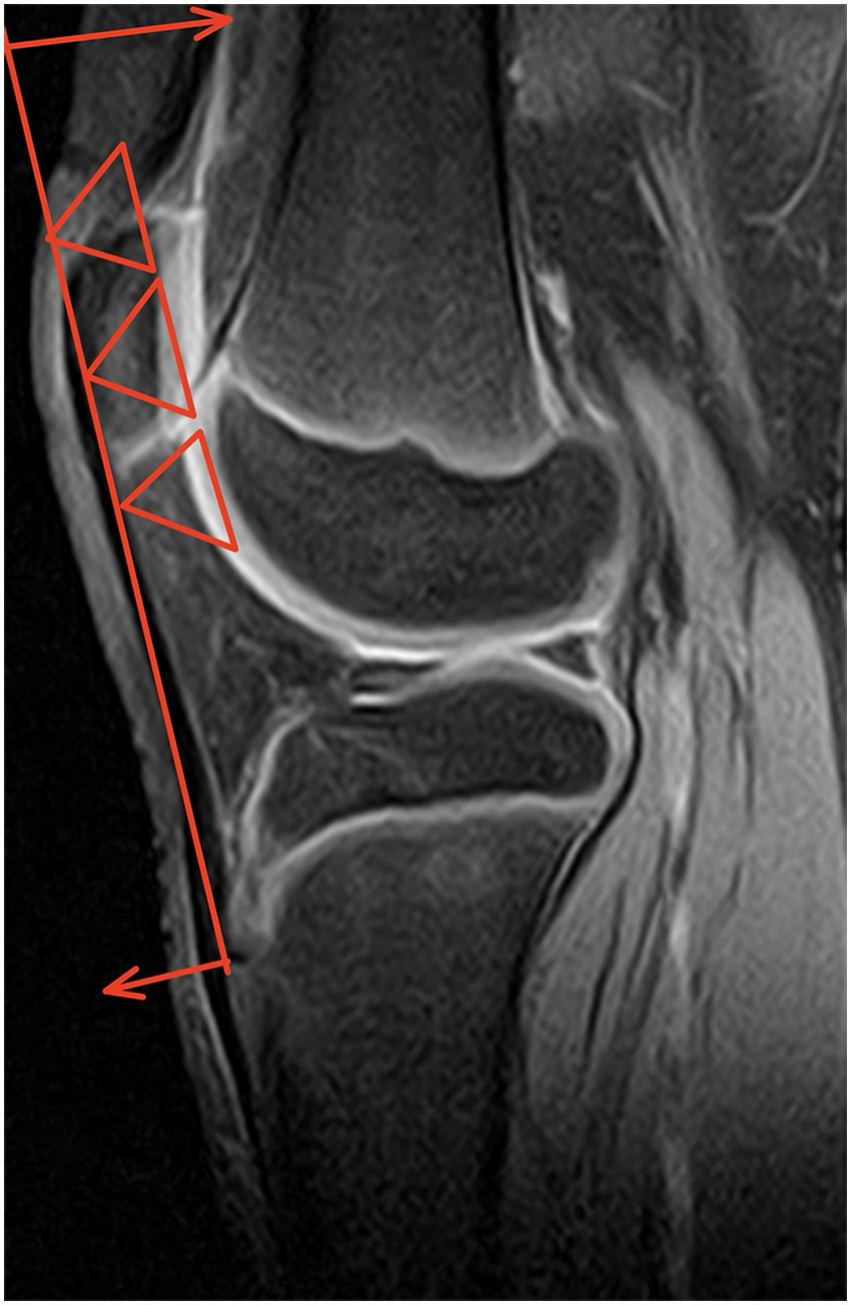
Schematic diagram of the knee joint lever biomechanical model for the tibial tuberosity apophysis in OSD patients.

Figures 2, 3 are schematic diagrams of the biomechanical model of the tibial tubercle apophysis. Figure 2 illustrates that as the patellar position progressively rises, the force F₂ increases with the widening angle between F₁ and F₂. Figure 3 depicts the force exerted on the tibial tubercle due to quadriceps contraction.

**Figure 4:**
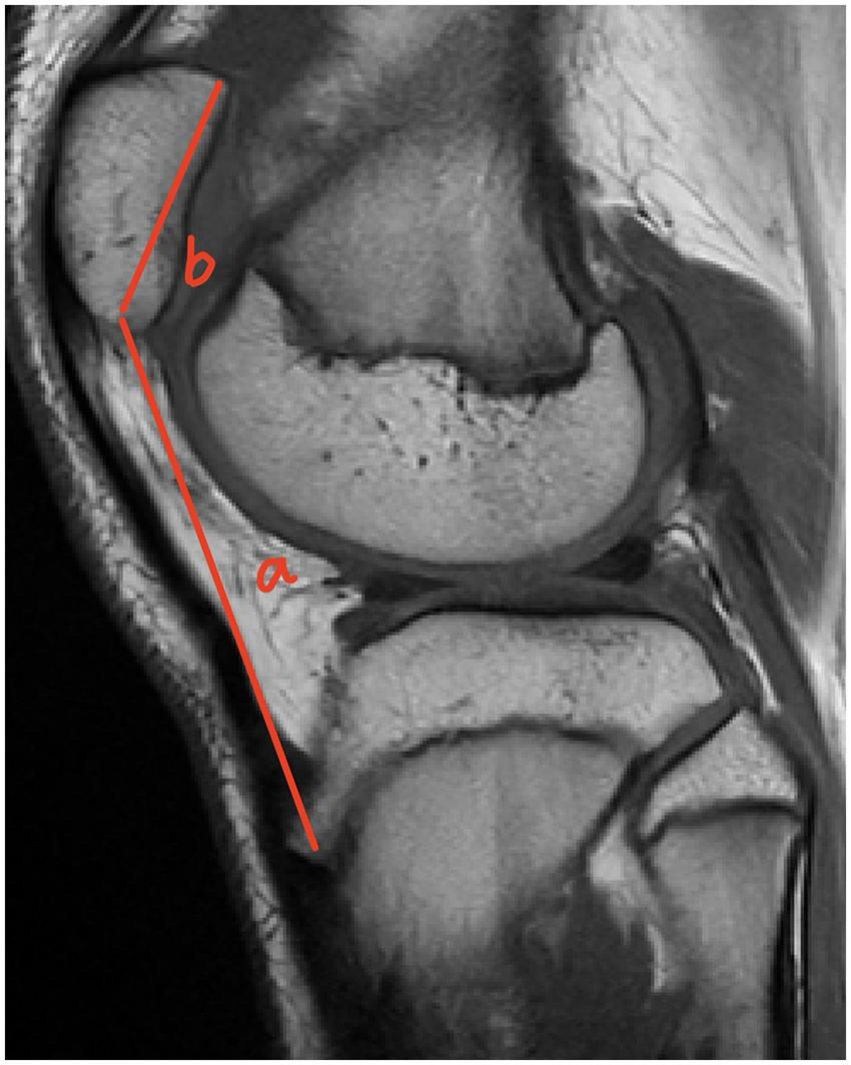
Sagittal MRI scan of the knee joint showing key anatomical measurements (a, b) for ISI calculation.

**Figure 5:**
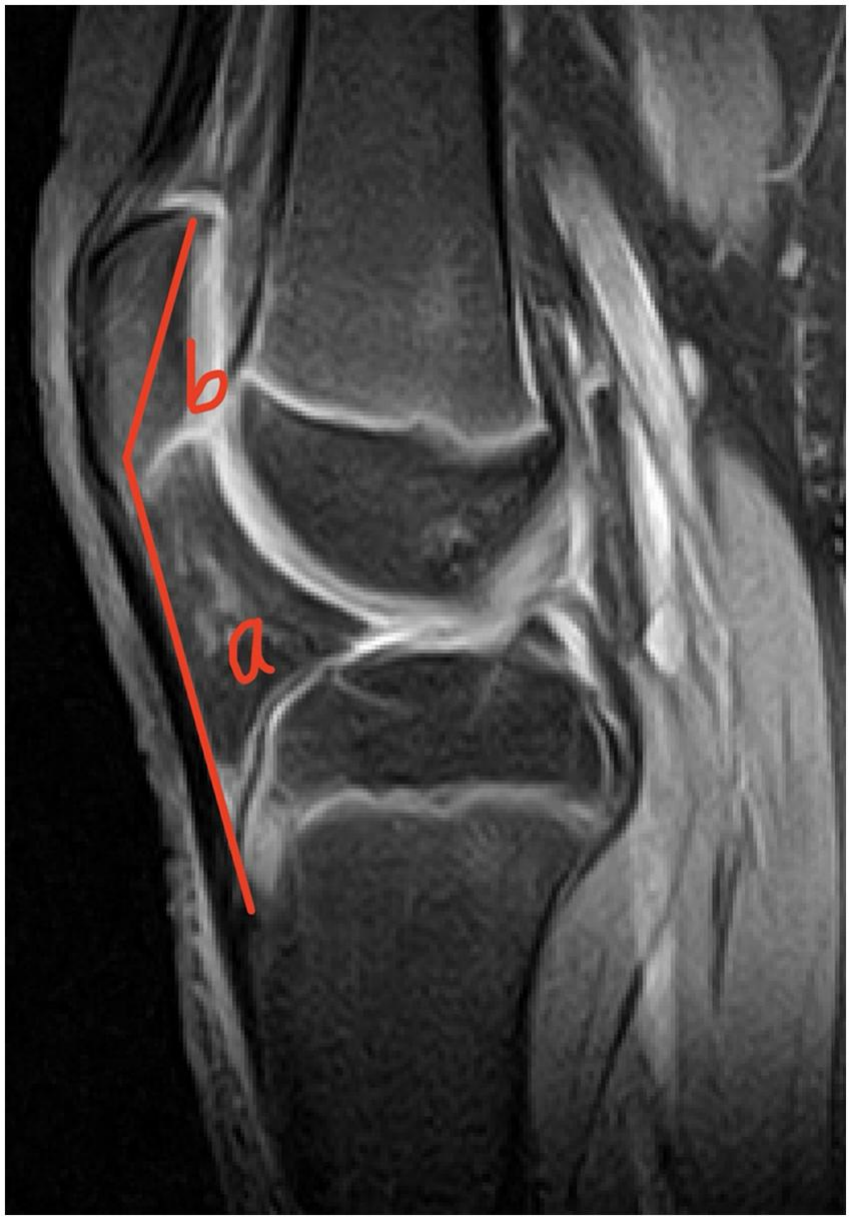
Sagittal MRI scan of the knee joint showing key anatomical measurements (a, b) for ISI calculation.

**Figure 6:**
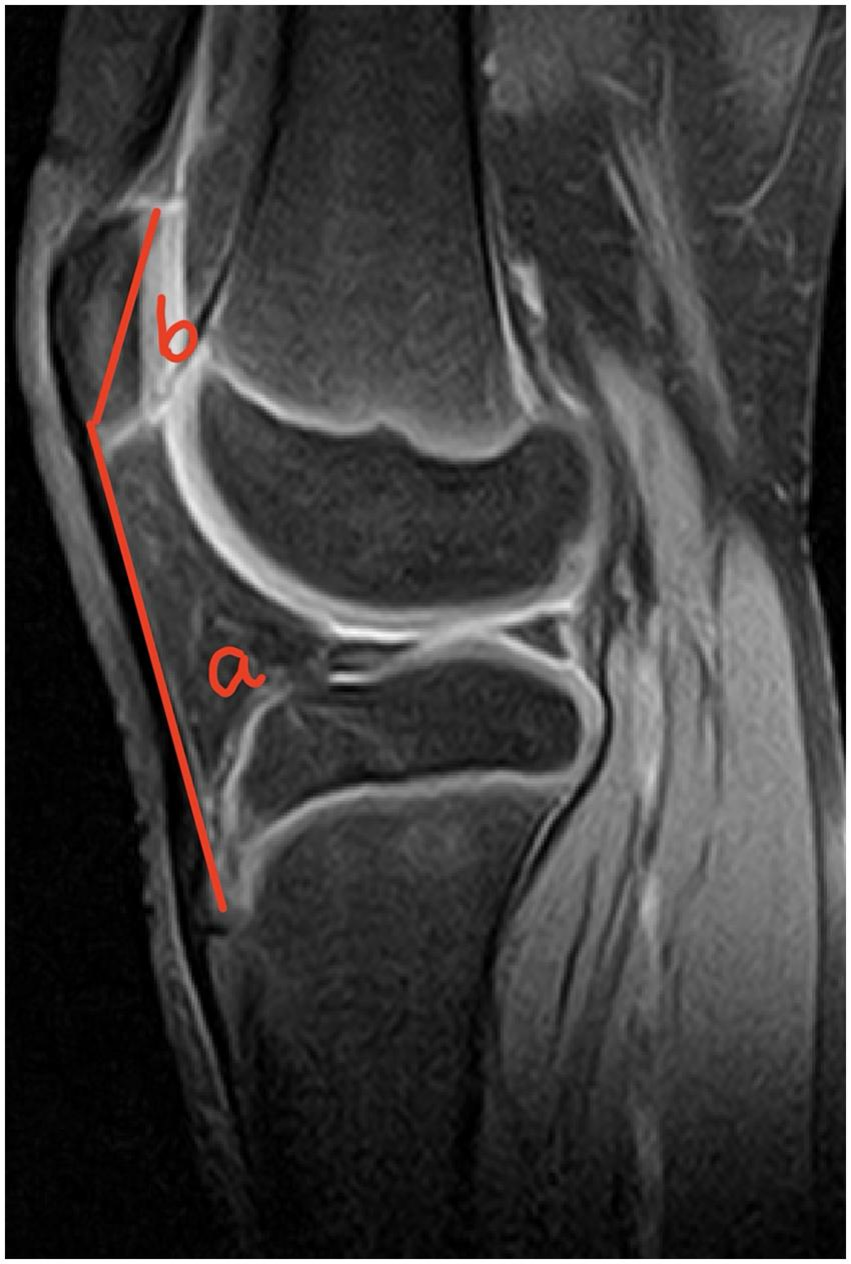
Sagittal MRI scan of the knee joint showing key anatomical measurements (a, b) for ISI calculation.

In Figures 4–6, the ratio b/a, representing the Insall-Salvati Index (ISI), is greater than 1.2 in all cases.

## 4 Discussion

OSD is a common cause of sports-related knee pain in adolescents. Its occurrence is closely related to damage of the tibial tuberosity apophysis during growth and development under repetitive traction, particularly in active adolescents aged 10–16 years (7). This study, analyzing 127 clinical cases, focused on the predictive value of BMI and the ISI for OSD onset. The core findings are discussed in two parts.

### 4.1 Association of ISI with OSD and biomechanical considerations

This study found that ISI has predictive ability for OSD onset, with an optimal cutoff value of 1.13, different from the traditional diagnostic value of 1.20 for patella alta. In terms of pathogenesis, patella alta is not the direct cause of OSD, consistent with the findings of Seyfettinoğlu et al. (4). However, from the perspective of the radiographic ISI measurement, this result indicates that ISI is not merely a morphological diagnostic indicator of patellar height but also a valuable early warning indicator for OSD risk. The core underlying mechanism involves biomechanical changes. The patella functions as a “fulcrum and pulley” in knee motion, and its height directly determines the length of the quadriceps moment arm and the tension in the patellar tendon. Mechanical model analysis (as shown in Figures 1, 2) indicates that the resultant force during knee extension and flexion is determined by the quadriceps contraction force (F1), the patellar tendon traction force (F2), and the angle θ (Figure 2) ($F\_\mathrm{resultant}=\sqrt{F1^{2}+F2^{2}+2F1F2cos\theta }$). When ISI value increases (i.e., the patella is positioned higher), it creates a dual effect: First, the angle θ increases, requiring the patellar tendon to withstand greater traction force (F2) to generate the same resultant force F. Second, the upward shift of the patella effectively lengthens the quadriceps moment arm, necessitating stronger quadriceps contraction force (F1), which is again transmitted via the patellar tendon as a stronger pulling force on the tibial tuberosity (Figures 1, 2). This sustained overload stress is the direct physical cause of apophyseal trauma, inflammation, and even avulsion fractures (8, 9). This study also revealed an interaction between BMI and ISI. ROC curve analysis for BMI showed an area under the curve of 0.793 (Figure 3). When the optimal BMI cutoff value was >23.9 kg/m^2^, OSD was more likely to occur. Although this value does not reach the diagnostic threshold for obesity, its proximity suggests that it is an important risk factor. High BMI not only directly increases the mechanical load on the knee joint (10) but is also often associated insulin resistance and a chronic inflammatory state, which may impair muscle repair capacity and stability (11, 12). Therefore, a high ISI value defines the abnormal direction of force application, while a high BMI increases the absolute magnitude of the force; their synergy significantly elevates the risk of OSD. Furthermore, this cross-sectional study found a significantly higher incidence in boys than girls, which is likely related to boys engaging more often in high-energy, high-volume sports (13) (regression analysis also indicated that a high exercise level was a factor influencing OSD onset), consistent with the results of Gaulrapp and Abou El-Soud (14). Paradoxically, a considerable proportion of children in the observation group developed OSD despite reporting lower exercise levels. Possible explanations include reduced daily activity in overweight children leading to decreased muscle strength, mass, and endurance, making them susceptible to high-intensity stimulation of the patellar ligament during occasional vigorous activities (e.g., physical education classes, necessary running, stair climbing). In addition, data showed that children with patella alta (ISI > 1.2) had higher pain scores, suggesting that ISI might also serve as a reference indicator for assessing disease complexity and prognosis, as the pain source might involve a mixture of OSD and patellofemoral instability, among other factors.

### 4.2 Clinical implications and study limitations

Based on the abovementioned mechanisms, the results of this study provide important insights for the clinical prevention and treatment of OSD. The implications are as following: (1) Early screening and prevention are essential. It is recommended to routinely perform X-ray screening and calculate the ISI for adolescent athletes, particularly those with obesity or a family history of OSD. An ISI > 1.14 should be considered a warning threshold. Even in clinically asymptomatic individuals, interventions can be initiated, including health education, guidance on scientific exercise, and focused strengthening of the quadriceps (particularly eccentric training) and hip muscles to enhance dynamic knee stability and biomechanically compensate for anatomical risk. (2) For the treatment of OSD, methods including rest, ice, stretching (15), physical therapy, topical non-steroidal anti-inflammatory drugs, and local injections of saline or dextrose (16, 17) can effectively relieve symptoms. However, immobilization remains the cornerstone of treatment. Studies indicate that this disease is not self-limiting (18), and immobilization during the acute phase is crucial for pain relief and healing. Traditional immobilization in extension fails to effectively restrict patellar movement, as isometric quadriceps contractions can still move the patella. This study provides a new perspective for immobilization during the acute phase: the use of a patellar distalization brace (designed to actively push the patella distally and fix it relatively horizontally via a knee-encircling strap), which could theoretically reduce patellar tendon tension at the source, creating a better healing environment for the tibial tuberosity apophysis, and potentially outperform traditional immobilization. For the minority of patients who do not respond to conservative treatment and have frequent dislocations complicated by severe patella alta, surgery remains the final option (10). However, timing must be strictly considered and surgery performed only after complete epiphyseal closure to avoid damaging the growth plate. The current research also has several important limitations that must be acknowledged. First, this is a cross-sectional study, which by design can only identify associations and not establish causality. The temporal relationship between ISI alterations and OSD onset remains unclear, and the possibility of reverse causality (i.e., that OSD-related anatomical changes might influence ISI measurements) cannot be ruled out. Second, a key methodological concern is the use of different imaging modalities between groups: The OSD group had ISI measured from various clinical images (DR, CT, or MR), while the control group underwent standardized lateral knee radiographs. Although our internal comparison suggested that measurement differences across these modalities were within an acceptable range (≤0.03), and inter- or intraobserver reliability was good, this inconsistency represents a potential source of measurement bias that should be considered when interpreting the results. Third, the sample size, particularly of the control group (*n* = 51), is relatively small compared to the observation group (*n* = 76), which may limit the statistical power and generalizability of the findings. Fourth, the classification of exercise levels relied on self-reported recall over the preceding week, which is susceptible to recall bias and reporting inaccuracies. Although we used a standardized classification based on WHO guidelines, this remains a limitation common to questionnaire-based assessments. Finally, the proposed ISI cutoff value of 1.14, while demonstrating excellent discriminative ability [area under the curve (AUC) = 0.933] in our cohort, lacks external validation. Its generalizability to other populations and settings needs to be confirmed in larger, prospective studies with standardized imaging protocols.

## 5 Conclusion

In summary, this study demonstrates that the ISI is a significant predictor of OSD in children, with an optimal cutoff value of 1.14. The pathogenesis of OSD results from the synergistic effect of biomechanical alterations due to abnormal patellar position (high ISI) and excessive mechanical load (high BMI and high exercise level). It is recommended to screen ISI in physically active adolescents, particularly boys and overweight individuals, for early risk warning and targeted interventions (such as quadriceps eccentric training). Future large-scale prospective studies are needed to validate this cutoff value and dynamically observe changes in ISI during the OSD course. In addition, optimizing acute phase immobilization protocols using biomechanically principled patellar distalization braces may improve patient outcomes.

## Data Availability

The raw data supporting the conclusions of this article will be made available by the authors, without undue reservation.
